# 80 Area Deprivation and Antibiotic Prescribing Patterns: Analysis of Medicare Part D Data and Socioeconomic Indices in Tennessee

**DOI:** 10.1017/ash.2026.10715

**Published:** 2026-06-23

**Authors:** Jonathan Shabab, Catherine Allan, Jessica Alban, Venkatraman Arakoni, Charles Foster

**Affiliations:** 1 Cleveland Clinic; 2 Cleveland Clinic Children’s

## Abstract

**Background:** Surgical Site Infection (SSI) surveillance in pediatric cardiac surgery requires an accurate denominator. Many programs use National Healthcare Safety Network (NHSN) selected CPT codes as these are available in real time. However, prior research demonstrates that these lists exclude many congenital operations, especially in infants. Society for Thoracic Surgery (STS) index procedures are comprehensive, but are not mapped to CPT codes and cannot drive routine surveillance. Objective: Develop and validate an expanded CPT code set that captures pediatric STS index cardiac procedures more completely than the NHSN CPT code set while maintaining specificity. **Methods:** We mapped STS pediatric index procedures performed at our institution during a derivation period to CPT codes. We restricted codes to cardiovascular procedures and organized additions into pediatric extensions of NHSN cardiac categories (P-CARD, P-CABG, P-PACE) plus a new arteries/veins group (P-ARTV). We then applied these codes to a second validation period to assess specificity. **Results:** The NHSN CPT set contains 176 codes. We added 62 CPT codes (P-CARD 50; P-ARTV 9; P-CABG 2; P-PACE 1). In the derivation period (2010–Feb 2022), 1,508 STS index cases were identified; 344 (25%) were not captured by the NHSN CPT set. Under-capture was greatest in infants (<1 year) with 242/724 (33.4%) missed, versus 102/784 (13.0%) missed in children (?1 year). Missed infant cases were associated with certain groups of procedures: aortic coarctation/arch/interrupted aortic arch (67/101, 66.3% expanded-only), pulmonary blood flow modification (62/66, 93.9% expanded-only), and single-ventricle staged palliation (62/128, 48.4% expanded-only) together accounted for 191/242 (78.9%) of missed cases. The standard NHSN set also captured only 1/11 patent ductus arteriosus procedures and none of vascular ring/aortopexy procedures (n = 5). NHSN capture was near-complete for conotruncal/right ventricular outflow tract repairs (119/120 infants; 106/109 ?1 year) and septal/atrioventricular canal repairs (139/139 infants; 124/125 ?1 year). In validation (Feb 2022–Mar 2024), the expanded set identified 317 cases; 258 were STS index (81.3%). Of 59 non-STS cases, 52 were captured by standard NHSN. The remaining 7 additional non-standard-NHSN, non-STS procedures were judged clinically relevant on review. **Conclusions:** The standard NHSN code set incompletely captures pediatric STS index procedures, especially procedures correcting congenital anomalies in infancy. A focused expansion of 62 CPT codes creates a more complete denominator to accurately monitor SSI rates.

